# Nephrogenic diabetes insipidus induced by ureter obstruction due to benign prostatic hyperplasia: A case report

**DOI:** 10.1097/MD.0000000000022082

**Published:** 2020-09-11

**Authors:** Hanyu Lou, Yimin Shen, Yi Xu, Wei Zhang, Yuezhong Ren

**Affiliations:** a Department of Endocrinology, The Second Affiliated Hospital of Zhejiang University School of Medicine, Zhejiang 310009; b Department of Cardiology, The Second Affiliated Hospital of Zhejiang University School of Medicine, Zhejiang 310009; c Department of Endocrinology, Zhejiang Provincial People's Hospital, Zhejiang 310003, China.

**Keywords:** nephrogenic diabetes insipidus, polyuria, prostatic hyperplasia, ureter obstruction

## Abstract

**Introduction:**

Diabetes insipidus can be a common cause of polyuria and hydronephrosis in the kidneys. However, there is few reported case of urinary obstruction induced nephrogenic diabetes insipidus.

**Patient concerns:**

A 60-year-old Chinese man came to our hospital with the complaints of polydipsia and polyuria for 1 month. His examination showed chronic kidney disease stage III with eGFR of 48.274 ml/min, and the plasma osmolality was 338.00 mOsm/(kg·H_2_O) with a urinary osmolality of 163.00 mOsm/(kg·H_2_O). Moreover, imagological examination of the urinary system showed benign prostatic hyperplasia and hydronephrosis.

**Diagnosis:**

He was considered with benign prostatic hyperplasia induced ureter hydronephrosis and nephrogenic diabetes insipidus.

**Interventions:**

He got the transurethral resection of the prostate to alleviate urinary retention.

**Outcomes:**

After that, the urine output gradually decreased, and the administered hydrochlorothiazide was stopped due to the improved renal function.

**Conclusion:**

Our study presents a case of nephrogenic diabetes insipidus caused by urinary obstruction. Differential diagnoses for diabetes insipidus as well as the relationship between nephrogenic diabetes insipidus and urinary obstruction are also considered in this study.

## 1 Introduction

Nephrogenic diabetes insipidus (NDI) is defined as the passage of large volumes (>3L/24 h) of dilute urine (<300 mOsm/kg). It is characterized by decreased ability to concentrate urine as a result of resistance to Arginine vasopressin(AVP) action in kidney.^[1]^ The majority of NDI are inherited, but the condition can be also acquired due to medications, biochemical influence, or even obstructive uropathy.^[1]^ The main strategy for NDI is to supply adequate fluid in combination with a low-salt and low-protein diet to minimize the obligatory water excretion. Thiazides and nonsteroidal anti-inflammatory drugs are the most common treatment for ameliorating the polyuric state in NDI.^[2]^ Diuretics in NDI patients reduce the urine output by promoting the reabsorption of sodium and water in the proximal tubule, thus delivering less water to the collecting ducts.^[3]^ Although these therapeutic approaches improve NDI symptoms, the urine concentrating defect is still considerable, making the patient's daily life in trouble. Previous study have demonstrated that figure out the potential cause, for example, to release the obstruction in obstructed kidney induced NDI can correct the polyuria syndrome.^[4–6]^ Therefore, it is significant to differentiate the polyuria related disease and explore their underlined cause. Here, we present a case diagnosed with NDI induced by urinary obstruction due to benign prostatic hyperplasia (BPH). The renal concentrating ability can be improved only after the resolution of urinary obstruction. In addition, the relationship between NDI and urinary obstruction, as well as other polyuria related disease are also discussed.

## 2 Case report

In January 2018, a 60-year-old Chinese man came to the hospital with complaints of thirst, polydipsia, and polyuria that had been occurring for 1 month. His symptoms began 1 month previously, when he started drinking 3 L of water daily, had an abnormal increase in the production of urine, and would urinate nearly every 15 minutes each day. There was no backache, blurred vision, or any weight loss. He had undergone treatment with Harnal and Finasteride for BPH for 8 years. Moreover, he experienced an increase in serum creatinine and chronic kidney disease stage III. There was no history of hypertension or cerebrovascular disease. A regular physical checkup before admission showed creatinine, 166 μmol/L (reference range, 40–106 μmol/L); urine specific gravity, 1.000 (reference range, 1.003–1.030); trioxypurine, 691 μmol/L (reference range, 208–428 nmol/L); serum sodium, 152.8 mmol/L (reference range, 135–145 nmol/L); potassium, 4.05 mmol/L (reference range, 3.5–5.5nmol/L); and hemoglobin A1c, 6.3%.

**Figure 1:**
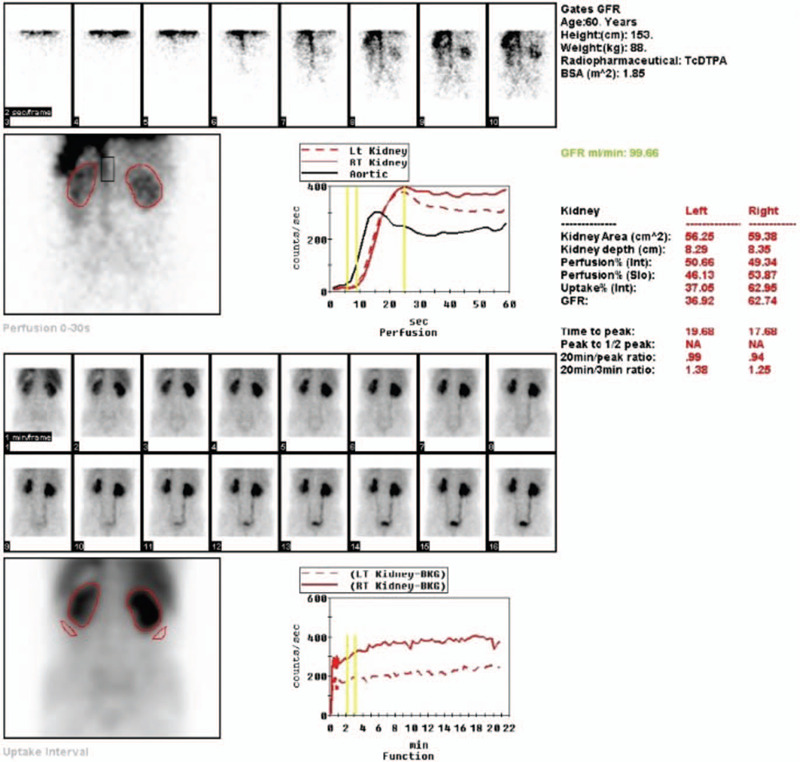
The dynamic renography of this patient.

At the time of admission, his BMI was 24.81 kg/m^2^. His blood pressure was 182/101 mmHg, with a pulse rate of 75/min. The physical examination was unremarkable. The laboratory results revealed the following results: blood urea nitrogen, 12 mmol/L (reference range, 2.8–7.2 mmol/L); creatinine, 173 μmol/L; serum sodium, 149.9 mmol/L; potassium, 3.83 mmol/L; and chloride, 115.0 mmol/L (reference range, 96–106 nmol/L). Microscopic examination of the urine revealed normal findings. The plasma osmolality was 338.00 mOsm/(kg·H_2_O), with a urinary osmolality of 163.00 mOsm/(kg·H_2_O), and the eGFR was 48.274 ml/min. Examination of cortisol, TSH, and FT4 was performed to detect other potentially associated pituitary disorders and presented normal results. Urinary system ultrasonography suggested prostatic hyperplasia and hydronephrosis. Computed tomography scans of the abdomen revealed hydronephrosis (pelvis and ureter). Renal dynamic imaging indicated blocked renal excretion abilities (Fig. 1).

**Figure 2:**
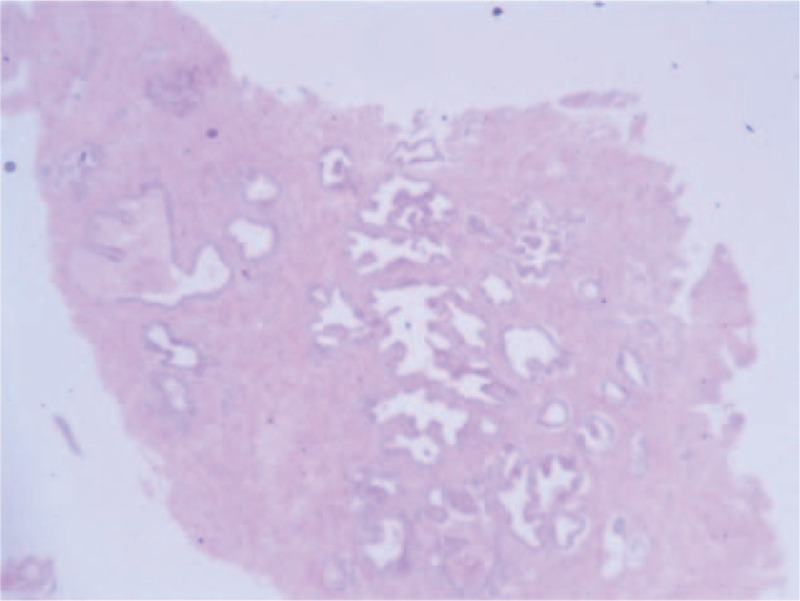
The pathological finding of benign prostatic hyperplasia.

**Figure 3:**
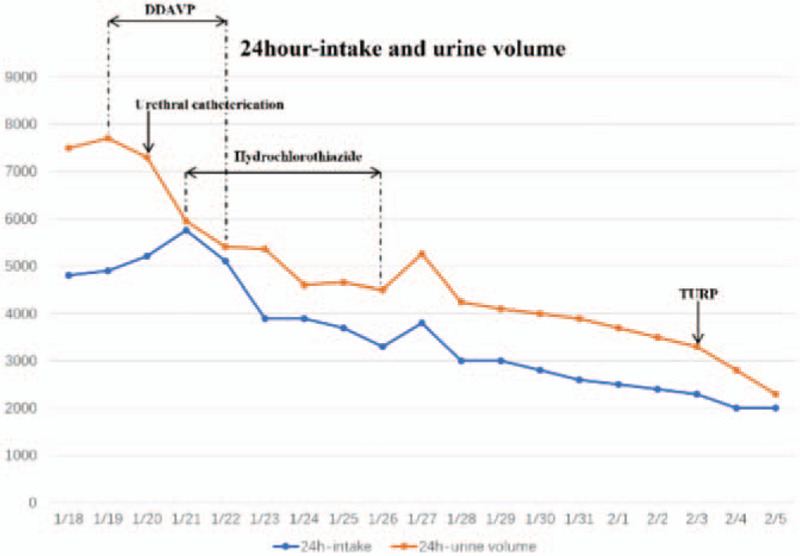
The 24 hour-intake and urine volume of this patient.

He was diagnosed with diabetes insipidus (DI). Later, he received desmopressin (DDAVP, Minirin) 0.05 mg once every 12 hours initially to treat DI and potassium chloride to correct his electrolyte abnormalities. However, the polyuria symptoms did not improve. Thus, five units of exogenous AVP were administered to identify the type of DI. Before, the plasma osmolality was 340.00 mOsm/(kg·H_2_O), with a urinary osmolality of 182.00 mOsm/(kg·H_2_O); 2 hours after administration, the plasma osmolality was 349.00 mOsm/(kg·H_2_O), with a urinary osmolality of 200.00 mOsm/(kg·H_2_O). Then, the patient was given hydrochlorothiazide (HCTZ) 12.5 mg twice a day, and the electrolyte levels become normal the next day. According to the results, the diagnosis of NDI could not be excluded. We considered that BPH related bilaterally obstruction of the ureter resulted in hydronephrosis and NDI. A catheter was used to alleviate urinary retention. After that, the urine output gradually decreased, and the hydrochlorothiazide was stopped due to the improved renal function. Before discharge, renal nuclide examination showed that the bilateral renal GFR was basically normal. His serum creatinine level returned to 108 μmol/L, and the patient was able to produce concentrated urine (plasma osmolality, 318.00 mOsm/(kg·H_2_O); his urinary osmolality was 302.00 mOsm/(kg·H_2_O)). Three days later, he got the transurethral resection of the prostate (TURP) in the urinary surgery department in our hospital (Fig. 2). Currently, no recurrence of polyuria has been complained for more than 12 months, indicating a feasible response to our therapeutic approach (Fig. 3).

## 3 Discussion

The patient presented here manifested with polyuria, polydipsia associated with hypernatremia in the presence of low-urine osmolality and high-serum osmolality. The desmopressin trials made urinary osmolality improved, but still at a lower level, thus, NDI rather than central diabetes insipidus (CDI) should be considered. The potential mechanism of polyuria could be attributed to the BPH induced urinary obstructive. Although he had a history of chronic nephrosis, the polyuria symptoms relieved when the hydronephrosis was resolved.

The standard criteria for polyuria is defined as a urine output of more than 3 L per day in adults.^[7]^ The differential diagnosis of polydipsia is extensive. There will be a few selected causes discussed as follows. NDI results from the failure of the kidneys to concentrate urine.^[8,9]^ Patients with this disease typically produce large quantities of diluted urine (up to 1 L/h in the most severe cases).^[10]^ The disease has a substantial impact on quality of life, as sleep is frequently interrupted. Treatment of acquired NDI should target the underlying cause, such as relief of any urinary obstruction.^[1]^ CDI is caused by the impaired production or secretion of vasopressin (VP) from the central nervous system.^[11]^ Known as a synthetic AVP V2R agonist, desmopressin has been used to treat CDI.^[12]^ VP by hypothalamic neurons in response to changes in soma volume is the key regulator of the water permeability of the collecting ducts.^[1]^ The water deprivation test and the DDAVP trial are essential for differentiating CDI and NDI.^[7]^ Moreover, psychogenic polydipsia (PPD) occurs in conjunction with chronic mental illness, especially schizoaffective disorders and schizophrenia.^[13]^ This condition presents with an excessive compulsive fluid intake without any potential medical cause, usually accompanied with headache, vomiting, lethargy, psychosis, and seizures.^[14]^ Fluid restriction has been the primary treatment for these patients.^[15]^

It is common that NDI can cause ureteral dilation, hydronephrosis, and urinary retention.^[16]^ However, in the case we presented here, BPH related ureteral dilation can also lead to NDI. Although these two diseases have similar clinical manifestations, their pathogenesis and primary treatments are different. NDI induced persistent large urine volumes can lead to urinary dilatation as well as the period of detrusor constriction that is not long enough to empty the large bladder, which results in further postvoiding residual volume (PRV).^[17]^ In this circumstance, impaired bladder contractility, diminished ureteric peristalsis, and large residual urine volumes worsen the obstruction and dilatation of the urinary tract, eventually resulting in renal failure.^[18,19]^ For the treatment, the primary step is to eliminate the underlying cause, for example, cession of lithium therapy.^[7]^ And thiazide diuretics, urethral catheterization, and transurethral incision of the bladder neck are available to reduce the PRV and relieve the urinary tract infections.^[17,19]^ As for the acquired NDI, it has been shown to occur both during a urinary obstruction as well as after the relief of a complete urinary obstruction.^[20]^ In cases of urinary tract obstruction, the natriuretic factors, such as atrial natriuretic peptide (ANP), may accumulate and lead to a reduced concentrating ability in the kidney. Moreover, the reduced ability of the thick ascending limb of Henle's loop to generate a concentrated interstitium and the collecting duct to increase water permeability in response to AVP can both induce acquired NDI.^[6]^ The obstruction caused hydronephrosis and polyuria can be improved only after the release of the urinary obstruction.

As for the treatment in this patient, a low dose of oral DDAVP was primarily given to manage polyuria, of which is a synthetic analog of the endogenous hormone AVP, but with a 2000–3000 fold lower vasopressor effect.^[21]^ The binding of desmopressin to the G protein-coupled V2 in the collecting duct results in activation of the aquaporin channels by adenylate cyclase increasing cAMP-dependent protein kinase and causing translocation of preformed aquaporin channels to the apical membrane, leading to increased water permeability and osmotic reabsorption of free water. And a lower dose of DDAVP based on the symptom control would avoid hyponatremia with excessive antidiuretic effects as well as allow normal drinking and full night's sleep.^[22]^ Later, HCTZ was prescribed into this patient after the diagnoses of NDI. Apart from its well-known capacity in the inhibition of the NaCl cotransporter in the distal convoluted tubule resulted water diuresis,^[23]^ Anne P. Sinke et al indicated that HCTZ partially protects polarized mouse cortical collecting duct (mpkCCD) cells against lithium-induced downregulation of aquaporin 2 (AQP2) abundance.^[24]^ However, whether HCTZ enhanced the regulation of AQP2 or improve the response to the AVP still need further examined.

## Author contributions

HYL and YMS wrote the manuscript; YX revised the manuscript; YZR and WZ reviewed and edited the manuscript.

## References

[1] Bockenhauer D Bichet DG Pathophysiology, diagnosis and management of nephrogenic diabetes insipidus. Nat Rev Nephrol 2015;11:576 88. 26077742 10.1038/nrneph.2015.89

[2] Gungor T Kokanaly MK Ozturkkan D A case of nephrogenic diabetes insipidus caused by partial bilateral ureteral obstruction due to advanced stage ovarian carcinoma. Arch Gynecol Obstet 2009;280:679 81. 19225793 10.1007/s00404-009-0987-2

[3] Wesche D Deen PM Knoers NV Congenital nephrogenic diabetes insipidus: the current state of affairs. Pediatr Nephrol 2012;27:2183 204. 22427315 10.1007/s00467-012-2118-8

[4] Hong EG Suh Y Chung YS A case of nephrogenic diabetes insipidus caused by obstructive uropathy due to prostate cancer. Yonsei Med J 2000;41:150 4. 10731936 10.3349/ymj.2000.41.1.150

[5] Kato A Hishida A Ishibashi R Nephrogenic diabetes-insipidus associated with bilateral ureteral obstruction. Internal Med 1994;33:231 3. 8069019 10.2169/internalmedicine.33.231

[6] Yoshioka K Imanishi M Sakai H Nephrogenic diabetes insipidus due to hydronephrosis in a patient with a solitary kidney. Clin Exp Nephrol 2003;7:243 6. 14586722 10.1007/s10157-003-0239-x

[7] Hui C Radbel JM Diabetes Insipidus. Treasure Island (FL): StatPearls; 2017.

[8] Morin D Vasopressin V2 receptor-related pathologies: congenital nephrogenic diabetes insipidus and nephrogenic syndrome of inappropiate antidiuresis. Nephrol Ther 2014;10:538 46. 25449762 10.1016/j.nephro.2014.09.002

[9] Namatame-Ohta N Morikawa S Nakamura A Four Japanese patients with congenital nephrogenic diabetes insipidus due to the AVPR2 mutations. Case Rep Pediatr 2018;2018:6561952. 30073107 10.1155/2018/6561952 PMC6057286

[10] Macau RA da Silva TN Silva JR Use of acetazolamide in lithium-induced nephrogenic diabetes insipidus: a case report. Endocrinol Diabetes Metab Case Rep 2018;2018:17 0154. 10.1530/EDM-17-0154 PMC5820740 29479446

[11] Sands JM Klein JD Physiological insights into novel therapies for nephrogenic diabetes insipidus. Am J Physiol Renal Physiol 2016;311:F1149 52. 27534996 10.1152/ajprenal.00418.2016 PMC5210200

[12] Lu HA Diabetes Insipidus. Advances in experimental medicine and biology 2017;969:213 25. 28258576 10.1007/978-94-024-1057-0_14

[13] Penders TM Stanciu CN Ganpat P Psychogenic polydipsia, hyponatremia and osmotic myelinolysis. BMJ Case Rep 2015;27:bcr2014207508. 10.1136/bcr-2014-207508 PMC4322270 25628321

[14] Unger M Xiong G Treatment of refractory psychogenic polydipsia with lithium. Ann Clin Psychiatry: Official Journal of the American Academy of Clinical Psychiatrists 2017;29:146 7. 28463347

[15] Ahmed SE Khan AH Acetazolamide: treatment of psychogenic polydipsia. Cureus 2017;9:e1553. 29021925 10.7759/cureus.1553 PMC5633260

[16] Ando F Mori S Yui N AKAPs-PKA disruptors increase AQP2 activity independently of vasopressin in a model of nephrogenic diabetes insipidus. Nat Commun 2018;9:1411. 29650969 10.1038/s41467-018-03771-2 PMC5897355

[17] Yoo TH Ryu DR Song YS Congenital nephrogenic diabetes insipidus presented with bilateral hydronephrosis: genetic analysis of V2R gene mutations. Yonsei Med J 2006;47:126 30. 16502494 10.3349/ymj.2006.47.1.126 PMC2687569

[18] Ando F Uchida S Activation of AQP2 water channels without vasopressin: therapeutic strategies for congenital nephrogenic diabetes insipidus. Clinical Exp Nephrol 2018;22:501. 10.1007/s10157-018-1544-8 PMC5956045 29478202

[19] Jin XD Chen ZD Cai SL Nephrogenic diabetes insipidus with dilatation of bilateral renal pelvis, ureter and bladder. Scand J Urol Nephrol 2009;43:73 5. 19037828 10.1080/00365590802580208

[20] Bockenhauer D van’t Hoff W Dattani M Secondary nephrogenic diabetes insipidus as a complication of inherited renal diseases. Nephron Physiology 2010;116:23 9. 10.1159/000320117 PMC3896046 20733335

[21] Di Iorgi N Napoli F Allegri AE Diabetes insipidus—diagnosis and management. Horm Res Paediatr 2012;77:69 84. 22433947 10.1159/000336333

[22] Oiso Y Robertson GL Norgaard JP Clinical review: treatment of neurohypophyseal diabetes insipidus. J Clin Endocrinol Metab 2013;98:3958 67. 23884783 10.1210/jc.2013-2326

[23] Miyoshi H Nakamura R Hamada H A Case of nephrogenic diabetes insipidus during transsphenoidal pituitary adenomectomy. J Neurosurg Anesthesiol 2016;28:79 80. 25844954 10.1097/ANA.0000000000000183

[24] Sinke AP Kortenoeven ML de Groot T Hydrochlorothiazide attenuates lithium-induced nephrogenic diabetes insipidus independently of the sodium-chloride cotransporter. Am J Physiol Renal Physiol 2014;306:F525 533. 24352504 10.1152/ajprenal.00617.2013

